# A Cluster-Randomised Trial of Staff Education to Improve the Quality of Life of People with Dementia Living in Residential Care: The DIRECT Study

**DOI:** 10.1371/journal.pone.0028155

**Published:** 2011-11-30

**Authors:** Christopher Beer, Barbara Horner, Leon Flicker, Samuel Scherer, Nicola T. Lautenschlager, Nick Bretland, Penelope Flett, Frank Schaper, Osvaldo P. Almeida

**Affiliations:** 1 WA Centre for Health and Ageing, Centre for Medical Research, Perth, Western Australia, Australia; 2 School of Medicine and Pharmacology, University of Western Australia, Perth, Western Australia, Australia; 3 Centre for Research on Ageing, Curtin University of Technology, Perth, Western Australia, Australia; 4 School of Psychiatry and Neurosciences, University of Western Australia, Perth, Western Australia, Australia; 5 Royal Freemasons, Victoria, Australia; 6 Department of Medicine, University of Melbourne, Melbourne, Victoria, Australia; 7 Academic Unit for Psychiatry of Old Age, St Vincent's Health, Department of Psychiatry, University of Melbourne, Melbourne, Victoria, Australia; 8 Rowethorpe Medical Centre, Bentley, Western Australia, Australia; 9 Brightwater Care, Perth, Western Australia, Australia; 10 Alzheimer's Australia WA Ltd, Perth, Western Australia, Australia; 11 Department of Psychiatry, Royal Perth Hospital, Perth, Western Australia, Australia; Georgetown University Medical Center, United States of America

## Abstract

**Background:**

The *Dementia In Residential care: EduCation intervention Trial* (DIRECT) was conducted to determine if delivery of education designed to meet the perceived need of GPs and care staff improves the quality of life of participants with dementia living in residential care.

**Methodology/Principal Findings:**

This cluster-randomised controlled trial was conducted in 39 residential aged care facilities in the metropolitan area of Perth, Western Australia. 351 care facility residents aged 65 years and older with Mini-Mental State Examination ≤24, their GPs and facility staff participated. Flexible education designed to meet the perceived needs of learners was delivered to GPs and care facility staff in intervention groups. The primary outcome of the study was self-rated quality of life of participants with dementia, measured using the QOL-Alzheimer's Disease Scale (QOL-AD) at 4 weeks and 6 months after the conclusion of the intervention. Analysis accounted for the effect of clustering by using multi-level regression analysis. Education of GPs or care facility staff did not affect the primary outcome at either 4 weeks or 6 months. In a *post hoc* analysis excluding facilities in which fewer than 50% of staff attended an education session, self-rated QOL-AD scores were 6.14 points (adjusted 95%CI 1.14, 11.15) higher at four-week follow-up among residents in facilities randomly assigned to the education intervention.

**Conclusion:**

The education intervention directed at care facilities or GPs did not improve the quality of life ratings of participants with dementia as a group. This may be explained by the poor adherence to the intervention programme, as participants with dementia living in facilities where staff participated at least minimally seemed to benefit.

**Trial Registration:**

ANZCTR.org.au ACTRN12607000417482

## Introduction

Population ageing is associated with an increase in the number of people living with chronic neuro-degenerative conditions, such as dementia. Australia has a complex system of care provision [1] and the majority of people with dementia live in the community. However, residential care is an important component of service delivery for older people with complex health problems, particularly severe dementia. It is thought that informed residential care can improve residents' quality of life. However, residential care providers face many challenges relating to physical environment, workforce and operational effectiveness. We found evidence of substantial perceived educational needs relating to the care of people with dementia among general practitioners (GPs) and residential care facility (RACF) staff [2]. In response, we developed a tailored educational intervention aiming to meet expressed needs of GPs and RACF staff [3]. Process evaluation indicated that the education interventions met the perceived learning needs of participants, however the effect of the intervention on the clinical outcomes of patients remained uncertain.

The *Dementia In Residential care: EduCation intervention Trial* (DIRECT) was conducted to determine if delivery of education designed to meet the perceived need of GPs and care staff improves the quality of life of people with dementia living in residential care facilities. We hypothesized that older adults with dementia living in RACF cared for by GPs and/or care staff randomly assigned to the intervention would have higher quality life scores by the end of six months than controls.

## Methods

### Ethics

The Human Research Ethics Committee at the University of Western Australia approved this study (RA 4/1/1685) and the study complied with the Helsinki Declaration for Human Rights. All GPs and RACF provided written agreement to participate in the study. Structured written and verbal consent procedures were used by research staff when approaching participants with cognitive impairment. The assent of “next of kin” was required for participation of people with cognitive impairment who were unable to provide informed consent. This trial was registered (ACTRN12607000417482) on 17/08/2007.

### Study Design and Setting

The protocol for this study has been described in detail previously [4] and is available as supporting information; see Protocol S1. The supporting CONSORT checklist is also available as supporting information; see Checklist S1. In brief, DIRECT was a prospective randomised controlled trial conducted in residential aged care facilities of the metropolitan area of Perth, Western Australia.

### Participants

351 permanent residents of aged care facilities aged over 65 years and with a Mini-Mental State Examination (MMSE) score ≤24, cared for by staff in 39 residential facilities and 55 GPs, were recruited between May 2007 and July 2008. Next-of-kin informants were available and agreed to participate for the majority of participants (n = 292; 83% at baseline) [5].

### Randomisation

Randomisation was carried out in a 2 by 2 factorial fashion. That is care facilities and GPs were independently randomised to intervention or control groups. Randomisation was carried out by a statistician not involved directly in the study. Thus, a participant with dementia could be cared for by a GP and RACF staff assigned to the intervention, either one of them assigned to the intervention, or neither of them assigned to the intervention. Data collection staff remained blind to facility and GP allocation.

### Intervention and control groups

A detailed qualitative research study was undertaken to determine the perceived needs of learners [2]. This informed development of an educational package. Development, and process evaluation of the education intervention has been described in detail previously [3]. Briefly, the main topics of the educational programs were communication, personal care and activities, positive values, behaviours of concern, pain management, the “3 Ds” (dementia, depression and delirium), and effective working between GPs and RACF. A flexible program for residential care facilities was developed in the format of brief thirty-minute blocks that could be combined in sessions of different lengths. The RACF program incorporated selection of local “dementia champions”, aiming to facilitate sustainable change at participating sites. For general practitioners, alternative face-to-face and self-directed packages were developed. The education program was delivered to intervention GPs and RACF staff between September 2008 and July 2009. Participants' evaluations were positive. For example, 1013 out of 1067 RCF staff feedback responses (95%) indicated that the session entirely met the participants' learning needs.

GPs and RACF staff assigned to the control group did not receive any specific intervention. The protocol did not preclude GPs and RACF staff assigned to the intervention or control groups independently accessing education, nor did we attempt to measure their participation in education other than that provided for the purposes of the study intervention.

### Outcomes

Primary and Secondary Outcomes were assessed at baseline and again 4 weeks and 6 months after the conclusion of the educational intervention.

#### Primary Outcome

The primary outcome of the study was the quality of life of the participants with dementia rated using the self-rated Quality of Life - Alzheimer's Disease Scale (QOL-AD) modified for use in long-term care settings [6], [7]. Higher scores on the QOL-AD indicate better quality of life (minimum 15, maximum 60). Research assistants were trained in the standard administration of assessment tools and adequate inter-rater reliability was established for the QOL-AD [8].

#### Secondary outcomes

Quality of life was also measured using the staff and next-of-kin rated QOL-AD and the Alzheimer Disease Related QOL Scale (ADRQOL) [9] which relies on caregiver interview. Higher scores on the ADRQOL (minimum 0, maximum 100) also indicate better quality of life. Informant ratings are required when the severity of a person's cognitive impairment precludes self rating. However, because informant ratings may differ from people's own ratings [5] of their quality of life, informant ratings were regarded as secondary outcomes. Family informants for the person with dementia (PWD) living in RACF were required to have visited the PWD on average at least once per week over the previous year. Staff informants were required to have known the resident for at least two weeks, and to have observed that resident at least 10 times, or for one hour in total, during the previous two weeks.

Other outcomes of interest were factors likely to impact on participants' quality of life including behavioural and psychological symptoms of dementia (measured with the Neuropsychiatric Inventory- NH version [10]), pain (measured using the Brief Pain Inventory modified verbal form [11] and PAIN_AD [12]), and use of physical restraint. Research staff recorded whether physical restraints were applied to the resident. This included fixed tray tables, “fall out” chairs and zipped bedding, as well as overt restraints.

### Survey of Attitudes

In addition, RACF staff attitudes were assessed at baseline, and again at conclusion of the educational intervention. The survey items were drafted by an experienced clinical psychologist (GJ, see acknowledgments) and were designed to measure changes in attitudes, knowledge, skills and care practice relating to the learning objectives of the educational intervention. Survey construction included some (reverse scored) negative items. A single page format was chosen, attempting to maximise convenience for participants. Piloting with carers sought to confirm potential for sensitivity to change; leading to some questions being discarded or modified. Successive iterations of the survey were piloted with 15 direct care RACF staff from culturally diverse backgrounds to ensure clarity of wording and readability. An optically scanable format of the survey was used, with individual numbering. We obtained the approximate number of employees from the RACF manager by telephone and then mailed surveys to the RACF manager for distribution, usually in the staff common room. The baseline survey was distributed to 1995 RACF staff in participating facilities. In addition, 1941 copies of the follow up survey were distributed to RACFs 6 weeks after conclusion of the education intervention. Surveys were anonymous, with addressed, stamped reply-paid envelopes included with each survey. Returned surveys were optically scanned and entered into an SPSS database. Items were scored using a 5-point scale ranging from ‘strongly agree’ to ‘strongly disagree’.

### Sample size and statistical analysis

The study was powered (power = 0.8, alpha = 0.05, two sided) to detect a 3.1 point difference (equivalent to approximately one half of one standard deviation in the population investigated) in QOL scores between intervention and control groups at six month follow –up (accounting for an estimated intra-class correlation of 0.05 and estimated cluster size of 9). The power calculation was reviewed prior to closing recruitment. It was confirmed that, because cluster size was smaller than anticipated (mean 5), recruitment could be closed. Analysis was by intention to treat and conducted using multilevel mixed-effects linear regression for each dependent variable (primary and secondary outcomes) in Stata version 11.0 (StataCorp, College Station, Texas). The effect of clustering by both facility and GP was accounted for by treating the facility and GP as random effects with GP nested within facility. For each outcome analysis, a model containing the GP intervention, the facility intervention, and the baseline values of outcome variables was used to estimate the marginal effect of each intervention. Next, the confounding effects of other covariates (MMSE, gender, age) were examined by comparing the adjusted and unadjusted intervention effects. Any covariates that produced clinically important changes in the intervention effect estimates were retained in the model. Secondary analyses were conducted to test the significance of any interaction between the facility and GP interventions (ie GP intervention group * RACF intervention group). Survey data were analysed using descriptive statistics. The proportion of highly positive responses (ie “strongly agree to positively worded items, and “strongly disagree” to reverse scored times) was compared between intervention and control groups using the chi-square (or Fisher Exact) test. Finally, to ensure that no potentially important associations had been overlooked, we also conducted alternative *post hoc* analyses. First, we examined the effect of the intervention on the quality of life of participants after excluding GPs who did not complete the face-to-face intervention and facilities in which fewer than 50% of staff had participated in any education sessions (i.e., analyses restricted to participants who adhered to the intervention). We also examined outcomes in a factorial fashion (that is for each of the four factorial intervention groups: control, GP education only, RACF staff education only, RACF staff and GP education). Main effects were examined, as well as a full factorial model including all interaction terms (ie GP intervention group * RACF intervention group, factorial intervention group * time). This analysis was conducted on an intention to treat basis, and then repeated after excluding GPs who did not complete the face-to-face intervention, and facilities in which fewer than 50% of staff had participated in any education sessions.

## Results

### Participants

**Figure 1:**
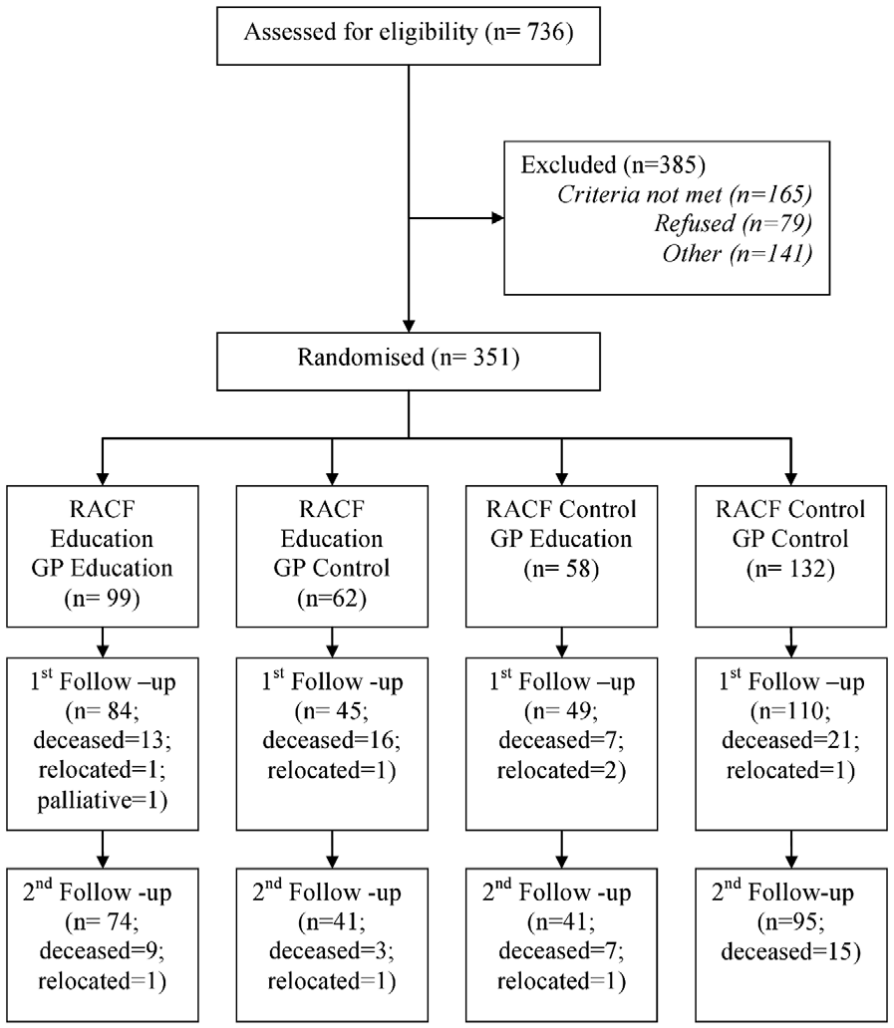
Study Flow Chart.

The study flow chart is shown in Figure 1. 351 participants with dementia living in 39 residential facilities and cared for by 55 GPs, were recruited between May 2007 and July 2008. Baseline demographic, clinical and quality of life data are summarised in Table 1. 288 participants with dementia were assessed in the first follow-up period, and 251 were assessed at second follow up. Subjects available at both follow up assessments had mean age of 85 years (SD 8 years), were predominantly (80%) female and had median MMSE score of 14 (IQR 5–19). Subjects lost to follow-up were more often male (35% cf 20%; p<0.01), took more medications (10 [8]–[13] cf 9 [7]–[12]; p = 0.02) and had lower staff-rated quality of life using both the QOL-AD (31±7 cf 33±8; p = 0.02.) and ADRQL (70±16 cf 74±16; p = 0.02).

**Table 1:** Demographic, clinical and quality of life data for participants at baseline.

	Usual care	RACF Intervention Only	GP Intervention Only	GP and RACF Interventions
Total n	132	62	58	99
Age (years)	84.4±8.1	86.1±8.4	84.6±8.8	86.4±6.6
Gender (Male)	35 (27%)	14 (23%)	15 (25%)	22 (22%)
MMSE, median (IQR)	12 (6–19)	10 (4–17)	16 (8–20)	15 (7–20)
Weight (kg)	62.3±14.4	60.4±13.5	64.0±14.2	62.6±14.2
Restraint observed	18 (14%)	8 (13%)	3 (5%)	9 (9%)
Perimeter secure (n)	61 (46%)	30 (48%)	18 (31%)	38 (38%)
Pain Reported (n)	17 (17%)	5 (10%)	14 (28%)	17 (18%)
Pain Observed (n)(PAIN-AD>1)	21 (16%)	3 (5%)	6 (11%)	12 (12%)
Self Rated QOL-AD	40±6	42±6	42±7	42±5
Staff Rated QOL-AD	31±8	33±8	34±6	32±8
NOK Rated QOL-AD	32±8	33±9	34±9	32±8
Staff Rated ADRQL	71.1±17.3	70.8±17.9	75.7±14.3	74.7±14.8
NOK Rated ADRQL	72.7±14.4	74.5±13.5	77.9±15.6	76.4±14.9
Medications (n)	10 (8–13)	10 (7–14)	9 (7–11)	9 (6–11)
10 Item NPI	14 (3–33)	2 (7–28)	12 (4–27)	12 (4–26)
12 Item NPI	15 (4–34)	18 (7–32)	16 (5–29)	12 (4–29)
10 Item NPI Distress	3 (0–11)	4 (0–9)	4 (0–10)	4 (1–9)
12 Item NPI Distress	4 (0–11)	5 (1–10)	4 (0–10)	4 (1–11)

### Adherence to the intervention

Sixteen of 27 GPs offered the dementia education participated. Two of the 16 GPs participated in both the face-to-face sessions as well as the self-directed learning package. Eighteen of 19 facilities offered the intervention participated and 326 RACF staff attended one or more of the 94 RACF education sessions. Overall, 29% of eligible RACF staff participated in the education sessions, but only 10% completed the entire educational program.

### Primary Outcomes

In the primary analyses (Table 2), GP intervention group did not influence participants' self-rated quality of life after four weeks (adjusted difference 0.33, 95%CI −1.89, 2.55) or six months of follow up (adjusted difference −0.61, 95%CI −3.07, 1.85). Similarly, facility staff intervention group did not influence participants' self-rated quality of life after four weeks (adjusted difference 1.12, 95%CI −1.11, 3.35) or six months (adjusted difference 0.97, 95%CI −1.55, 3.50). Informant rated quality of life was also not influenced by randomisation of GPs or RACF staff to the education intervention groups.

**Table 2:** Adjusted quality of life among control and intervention groups (and adjusted differences): ‘intention to treat’ analysis.

	RACF Staff Education			GP Education		
	Control	Education	Adjusted Difference (95% CI)	Control	Education	Adjusted Difference (95% CI)
*Four week follow up*						
Self rated QOL-AD	40.66	41.78	1.12 (−1.11, 3.35)	41.00	41.34	0.33 (−1.89, 2.55)
Staff rated QOL-AD	35.03	33.26	−1.77 (−4.35, 0.81)	34.65	33.87	−0.78 (−3.05, 1.48)
NOK rated QOL-AD	31.58	31.72	0.14 (−1.56, 1.84)	31.42	31.92	0.49 (−1.20, 2.19)
Staff rated ADRQL	72.37	73.12	0.75 (−2.63, 4.13)	72.85	72.54	−0.30 (−3.66, 3.06)
NOK rated ADRQL	74.39	74.87	0.49 (−3.00, 3.98)	74.84	74.31	−0.54 (−4.04, 2.96)
*Six month follow up*						
Self rated QOL-AD	41.68	42.65	0.97 (−1.55, 3.50)	42.45	41.84	−0.61 (−3.07, 1.85)
Staff rated QOL-AD	33.92	32.74	−1.18 (−3.64, 1.28)	32.78	34.36	1.58 (−0.66, 3.82)
NOK rated QOL-AD	31.73	30.67	−1.07 (−3.34, 1.21)	31.27	31.20	−0.07 (−2.31, 2.17)
Staff rated ADRQL	70.44	69.83	−0.61 (−5.23, 4.01)	70.55	69.69	−0.85 (−5.08, 3.38)
NOK rated ADRQL	74.06	72.14	−1.92 (−6.15, 2.32)	72.65	73.67	1.02 (−3.23, 5.27)

### Secondary Outcomes

The majority of the secondary outcomes were not influenced by random assignment to the GP or RACF intervention groups (Table 3). Restraint was observed less frequently in the GP intervention group after four weeks (OR 0.22, 95%CI 0.098, 0.54) and documented less frequently in the GP intervention group after six months (OR 0.13, 95%CI 0.03, 0.47). Pain was observed less frequently in the GP intervention group after four weeks (OR 0.31, 95%CI 0.13, 0.75). However results regarding documented pain assessment were mixed, with an increase in the RACF intervention group after six months (OR 3.75, 95%CI 1.26, 11.14) but a decrease in the GP intervention group (OR 0.36, 95%CI 0.14, 0.89). After six months, case conferences were documented more frequently in the RACF intervention group (OR 4.08, 95%CI 1.42, 11.67), but comprehensive medical assessments were documented less frequently in the GP intervention group (OR 0.19, 95% 0.06, 0.62).

**Table 3:** Adjusted secondary outcomes among control and intervention groups (and adjusted OR); ‘intention to treat’ analysis.

	RACF Staff Education			GP Education		
	Control	Education	Adjusted OR(95% CI)	Control	Education	Adjusted OR(95% CI)
*Four week follow-up*						
Restraint observed (%)	18.85	15.68	1.03 (0.39, 2.78)	29.88	12.19	**0.22 (0.09, 0.54)**
Restraint document (%)	14.01	13.06	1.14 (0.31, 4.22)	16.37	10.46	0.40 (0.14, 1.20)
Brief Pain Inventory (%)	22.43	22.22	1.75 (0.75, 4.08)	27.45	16.84	**0.31 (0.13, 0.75)**
Pain assess documented (%)	57.89	54.12	0.88 (0.20, 3.83)	58.04	51.48	0.82 (0.29, 2.29)
Hospital presn 30 d (%)	5.01	3.70	0.62 (0.18, 2.12)	4.92	5.42	1.34 (0.40, 4.47)
Case conference (%)	8.42	26.08	**4.08 (1.42, 11.67)**	10.58	17.91	1.59 (0.64, 3.95)
CMA (%)	21.66	18.42	1.61 (0.58, 4.50)	24.45	16.15	0.84 (0.33, 2.15)
GP review 30 d (%)	68.12	74.29	1.22 (0.37, 4.06)	70.25	70.16	0.78 (0.30, 2.03)
NPI	33.46	35.77	0.94 (0.46, 1.93)	36.32	33.49	0.75 (0.37, 1.54)
NPI – Distress	22.83	27.70	0.71 (0.27, 1.85)	21.85	26.66	1.41 (0.65, 3.06)
*Six month follow-up*						
Restraint observed (%)	23.57	23.69	1.06 (0.39, 2.94)	29.35	20.62	0.44 (0.17, 1.11)
Restraint document (%)	18.34	12.35	1.53 (0.33, 7.14)	26.30	7.32	**0.13 (0.03, 0.47)**
Brief Pain Inventory (%)	16.87	23.68	1.98 (0.81, 4.83)	21.25	18.99	0.60 (0.25, 1.47)
Pain assess documented (%)	47.18	73.59	**3.75 (1.26, 11.14)**	67.09	44.70	**0.36 (0.14, 0.89)**
Hospital presn 30 d (%)	3.68	3.48	0.97 (0.24, 3.99)	3.68	3.48	0.95 (0.23, 3.93)
Case conference (%)	5.65	19.83	3.23 (0.95, 11.01)	10.63	14.26	1.02 (0.34, 3.02)
CMA (%)	5.96	15.33	1.83 (0.60, 5.61)	18.10	7.42	**0.19 (0.06, 0.62)**
GP review 30 d (%)	62.86	78.05	2.17 (0.94, 5.02)	69.64	69.15	0.89 (0.40, 1.96)
NPI	27.07	32.92	1.18 (0.56, 2.49)	32.59	30.57	0.81 (0.40, 1.61)
NPI – Distress	12.96	22.50	1.17 (0.40, 3.41)	10.83	25.42	1.66 (0.63, 4.35)

### Survey of RACF staff

There were 450 responses (approx 23% of maximum response rate possible) to the baseline survey and 398 responses (approx 21% of maximum response rate possible) to the follow-up survey of RACF staff. Personal care delivery staff were the largest group of respondents (50% of the baseline survey, and 55% of the follow-up survey; Table 4). They were typically 46 to 55 years old and had worked in aged care for more than five years. Baseline responses were largely positive (Table 5). At follow-up, the proportion of intervention group participants responding strongly positively did not decrease for any items, and increased for four items.

**Table 4:** Demographics of RACF survey respondents.

	Baseline n = 450	Post-intervention n = 398
Length of service, years median (IQR)	7 (3,14)	8 (3, 17)
*Role n(%)*		
Carer	219 (50%)	207 (55%)
Enrolled Nurse	33 (8%)	38 (10%)
Registered Nurse	48 (11%)	44 (12%)
Manager	22 (5%)	14 (4%)
Physiotherapist	9 (2%)	7 (2%)
OT	12 (3%)	7 (2%)
Therapy Assistant	46 (10%)	24 (6%)
Other	48 (11%)	33 (9%)
*Age n(%)*		
<25	33 (8%)	30 (8%)
26–35	43 (10%)	48 (13%)
36–45	85 (20%)	58 (15%)
46–55	166 (38%)	150 (39%)
56–65	98 (23%)	85 (22%)
65+	8 (2%)	10 (3%)

n = total number of surveys (including incomplete); % = % of valid responses.

**Table 5:** Proportion of highly positive responses to survey items (Control facilities compared to intervention facilities, at baseline and follow up 6 weeks after conclusion of the education intervention).

	Baseline			Follow-up		
Survey Item (R = reverse scored)	Control	Intervention	p	Control	Interventn	p
1. I try to understand what a resident with dementia is experiencing.	101 (48%)	123 (51%)	.507	83 (47%)	119 (55%)	.132
2. A person with dementia can feel happy no matter how far the dementia has progressed.	53 (25%)	68 (28%)	.460	42 (24%)	68 (31%)	.109
3. There is no need for me to think about negative public attitudes towards dementia. (R)	32 (15%)	33 (14%)	.652	38 (22%)	41 (19%)	.580
4. Visiting doctors and staff at my workplace work well together to reduce pain in residents with dementia.	66 (31%)	73 (31%)	.934	49 (28%)	81 (38%)	**.047**
5. As a rule staff replace the family in all the caring for a new resident with dementia. (R)	24 (11%)	23 (10%)	.552	8 (5%)	21 (10%)	.054
6. Depression and withdrawal are always part of the stages of dementia. (R)	26 (12%)	17 (7%)	.066	7 (4%)	12 (6%)	.480
7. Showering is the only way to keep residents really clean. (R)	14 (7%)	11 (5%)	.336	25 (14%)	49 (23%)	**.039**
8. The main reason for doing activities with residents with dementia is to keep their skills up. (R)	25 (12%)	32 (13%)	.653	16 (9%)	20 (9%)	.968
9. I welcome family members who want to do some of the personal care for their resident with dementia.	79 (38%)	71 (30%)	.081	64 (37%)	77 (36%)	.810
10. Some residents with dementia fear running water on their skin.	28 (13%)	35 (15%)	.705	29 (17%)	50 (23%)	.107
11. Dementia dulls sensitivity to pain and so pain is less of an issue with residents with dementia. (R)	12 (6%)	8 (3%)	.229	66 (38%)	92 (43%)	.328
12. I learn most about the well-being of a resident with dementia by watching their face and actions.	60 (29%)	65 (27%)	.746	40 (23%)	62 (29%)	.185
13. Residents and families have a say about what medications the resident will be given.	24 (11%)	22 (9%)	.429	21 (12%)	24 (11%)	.794
14. Management at my workplace is more interested in money than people. (R)	11 (5%)	10 (4%)	.583	45 (26%)	80 (38%)	**.011**
15. When a resident suddenly has times of clarity and confusion in the same day, it shows a change in their stage of dementia. (R)	7 (3%)	3 (1%)	.199	8 (5%)	12 (6%)	.656
16. I am able to help residents with dementia experience less pain.	25 (12%)	25 (11%)	.609	23 (13%)	41 (19%)	.103
17. Staff at my workplace do not have a say on decisions about residents with dementia. (R)	7 (3%)	2 (1%)	.089	27 (15%)	49 (23%)	.071
18. Staff can often disagree among themselves as to whether a behaviour stresses them or not.	13 (6%)	16 (7%)	.820	13 (8%)	15 (7%)	.839
19. Most behaviours of concern are caused by something done with the person just before the behaviour starts. (R)	9 (4%)	12 (5%)	.715	7 (4%)	6 (3%)	.536
20. Good design of the care environment can prevent some behaviours of concern.	53 (25%)	59 (25%)	.870	42 (24%)	49 (23%)	.779
21. Ignoring the behaviour is a good way to teach a resident that the behaviour is not wanted. (R)	5 (2%)	5 (2%)	1.000	60 (34%)	86 (40%)	.264
22. Management at my workplace support me to work well with residents with dementia.	66 (31%)	78 (33%)	.808	47 (27%)	85 (39%)	**.013**
23. Restraint is good for residents with dementia who are likely to fall when trying to stand. (R)	13 (6%)	8 (3%)	.152	37 (21%)	51 (24%)	.532
24. Visiting doctors and staff at my workplace work well together to improve the quality of life of residents with dementia.	74 (35%)	75 (31%)	.370	52 (30%)	91 (42%)	**.010**

### Post hoc analysis according to participation in the education intervention and factorial group

After excluding facilities in which fewer than 50% of staff had participated in any education sessions, the RACF education intervention was associated with a difference of 6.14 points in self-rated quality of life of participants (95%CI 1.14, 11.15; Table 6). Excluding GPs who had attended fewer than 50% of the education intervention did not produce any significant differences in participant quality of life outcomes associated with the GP education intervention.

**Table 6:** *Post hoc* analysis: Adjusted quality of life among control and intervention groups (and adjusted differences) at four weeks and six months, according to participation in the education intervention.

	RACF Staff Education			GP Education		
	Control	Education 50%+ participants	Adjusted Difference (95% CI)	Control	Education 50%+ attendance	Adjusted Difference (95% CI)
*Four week follow-up*						
Self rated QOL-AD	39.83	45.97	**6.14 (1.14, 11.15)**	40.58	40.03	−0.55 (−4.23, 3.13)
Staff rated QOL-AD	35.37	31.91	−3.46 (−8.68, 1.76)	34.79	33.09	−1.70 (−5.00, 1.16)
NOK rated QOL-AD	31.29	31.89	0.59 (−3.14, 4.33)	31.14	31.47	0.32 (−2.33, 2.98)
Staff rated ADRQL	71.44	70.81	−0.63 (−8.64, 7.38)	72.20	69.32	−2.88 (−7.74, 1.97)
NOK rated ADRQL	73.37	74.74	1.36 (−5.87, 8.60)	74.09	71.70	−2.39 (−7.76, 2.97)
*Six month follow-up*						
Self rated QOL-AD	40.57	45.68	5.11 (−0.58, 10.81)	42.11	40.78	−1.33 (−5.01, 2.34)
Staff rated QOL-AD	33.39	32.85	−0.54 (−5.74, 4.66)	32.68	33.58	0.90 (−2.21, 4.02)
NOK rated QOL-AD	31.52	33.86	2.34 (−4.12, 8.81)	31.30	32.78	1.47 (−2.23, 5.17)
Staff rated ADRQL	69.6	71.74	2.14(−6.84, 11.11)	69.61	66.66	−2.95 (−8.10, 2.21)
NOK rated ADRQL	73.02	75.32	2.30 (−8.86, 13.46)	72.39	73.27	0.88 (−6.06, 7.82)

**Figure 2:**
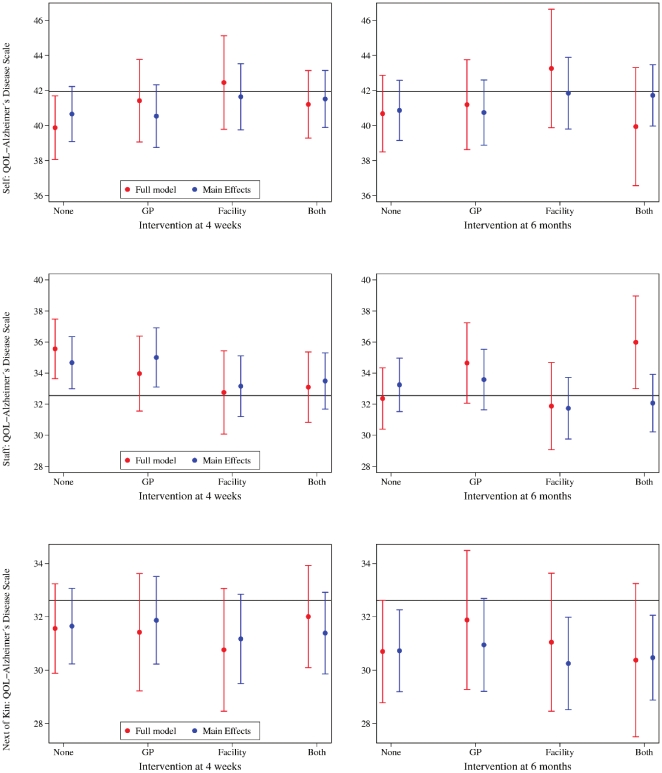
The figures show the effect of the intervention on quality of life scores according to patients (self), staff and next of kin 4 weeks and 6 months after the intervention. The horizontal reference line represents the mean score at baseline. The circles depicts the mean score by group including all interactions, and after excluding group interactions. The vertical whiskers represent the 95% confidence interval of the mean score.

**Figure 3:**
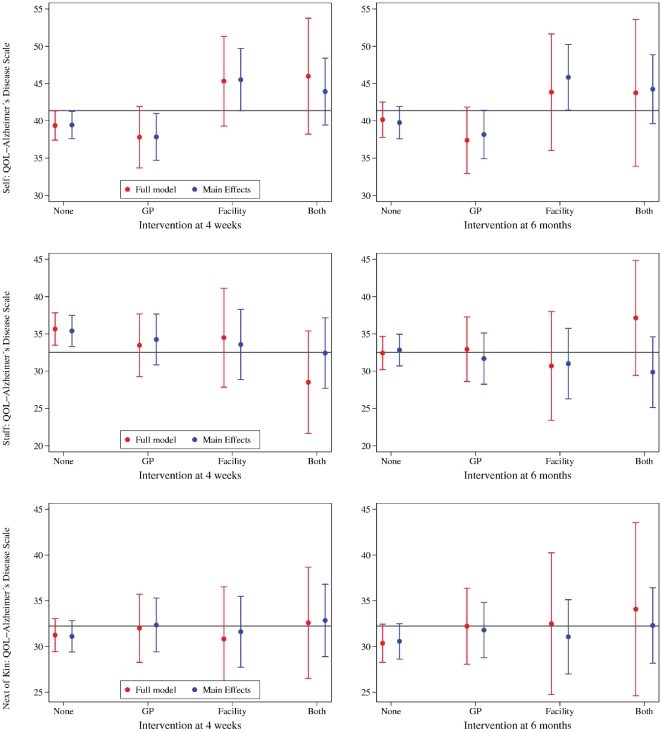
The figures show the effect of the intervention on quality of life scores according to patients (self), staff and next of kin 4 weeks and 6 months after the intervention. The results exclude participants whose GPs did not complete the face-to-face education or whose facilities had less than 50% of staff taking part in the educational program. The horizontal reference line represents the mean score at baseline. The circles depict the mean score by group including all interactions, and after excluding group interactions. The vertical whiskers represent the 95% confidence interval of the mean score.

Results for each of the four factorial groups (control, GP education only, RACF staff education only, RACF staff and GP education) are shown in Figure 2 (intention to treat analysis) and Figure 3 (after exclusion of GPs who did not complete the face to face intervention, and facilities in which fewer than 50% of staff had participated in any education sessions). A trend to improved quality of life in the RACF intervention and combined intervention groups was present after excluding non-adherent GPs and RACF staff, although these remained of borderline statistical significance.

## Discussion

### Main findings and implications

Delivery of the DIRECT education intervention to general practitioners and care facility staff did not improve the quality of life ratings of people with dementia living in residential care facilities. Low rates of participation in the education intervention may explain this finding, given that improvements in self-rated quality of life were present among participants in facilities where more than 50% of staff attended at least one education session. In contrast, participation by GPs did not appear to influence QOL, even after excluding GPs who attended fewer than 50% of education intervention sessions. Analysis of secondary outcomes, and survey of the attitudes of care staff, suggests that some care practices may have changed, and staff attitudes improved, as a result of the intervention. Restraint, which is an important secondary variable relevant to the quality of life of people with dementia living in residential care [5], appeared to have been impacted by the GP intervention. Pain was also reported less frequently among participants with dementia in the GP intervention group after four weeks, although documentation of pain assessment also decreased in this group. These findings suggest persisting problems with documentation of pain assessment, which has long been recognised in residential care settings [13].

The DIRECT intervention was conceived with extensive input from participants and explicitly designed to, where possible, overcome barriers to participation and sustainability [3]. However, there are many external constraints in the aged care sector that could not be addressed in design of the intervention. Most obvious are funding constraints, implying that future interventions should consider budgeting for staff backfill, which may facilitate staff participation in education programs.

### Findings in context of other studies

A 6 month training and education intervention, enrolling residents who staff perceived as difficult to care for because of behavioral challenges, was associated with small improvements in scores for depression and cognitive impairment (but not for behavior rating or functional status) [14]. This intervention comprised seven one-hour seminars delivered by hospital outreach staff. Enrolment of a broad range of participants with dementia in the DIRECT study, and use of a quality of life primary outcome, may explain the contrasting findings of the two studies.

There are several alternatives to education to improve the lives of people with dementia living in residential care. A personalized liaison intervention did not significantly reduce total unmet needs relative to the control group [15]. Implementation of person centred care and dementia care mapping reduced agitation among people with need-driven dementia compromised behaviours [16]. For the person centered care intervention, one investigator provided training and support (assisting staff to develop and implement care practices for 29% of the residents), supplemented by telephone contact. Resident agitation, perceived by staff, improved but quality of life did not.

### Strengths and limitations

To our knowledge the detailed attempt to respond to the perceived needs of learners (both GPs and RACF staff) is unique. The education intervention included strategies to facilitate sustainability and overcome barriers to participation in the residential cares setting (such as flexible deign and delivery; and nomination of, and support for, ‘dementia champions’) and was consistent with current recommendations for education in long term care [17]. Participants perceived the education programs developed as meeting their needs. Despite these factors, overall attendance remained low, emphasising the difficulty of delivering education in the residential care sector.

This study was moderately large, randomized, and comprised prolonged and detailed follow up. Participants lost to follow-up (largely due to mortality) tended to take more medications and had lower quality of life at baseline, so may have been a sicker group. The primary analysis was conservative (controlling for baseline values of the variables of interest). However, time was not included as a main effect in our statistical models, although an important effect of time was excluded in *post hoc* secondary analyses. A person-centered approach was adopted, in which self-rated quality of life was regarded as the primary outcome of interest. In addition, quality of life was measured comprehensively, by use of two informant scales. These scales are considered valid, but the possibility of relevant unmeasured effects of the intervention is not completely excluded. A few subjects, in both groups, may have declined cognitively to a state where they were unable to complete the QOL-AD reliably, which may have diluted the treatment effect. However this measure is robust even in the presence of moderately severe dementia [6] and this is unlikely to have influence the results to any degree. Various secondary outcomes, as well as the attitudes of staff, were measured. The post hoc analyses should be regarded as hypothesis generating as they were not pre-specified, and are subject to biases (for example, facilities in which a majority of staff participated in education intervention sessions may differ systematically from those in which participation of the majority of staff was not be achieved). In addition, some caution is justified in relation to potential generalisability of the data. For example, we adopted a pragmatic approach to recruitment of people with cognitive impairment and did not validate the diagnosis of dementia.

### Conclusion

These data suggest that despite detailed development and implementation of an education intervention designed to meet the perceived needs of learners, overall participation rates in the education intervention remained low, and measurable improvements in the quality of life of participants with dementia were not associated with the intervention. These data emphasize the importance of evaluating outcomes resulting from education delivery (in addition to learner's perceptions). Future studies should further evaluate the barriers to staff participating in education programs. In future studies of educational interventions, funding may need to be made available to ensure that the majority of staff are given an opportunity to complete the education. In addition, future work should evaluate alternative models of quality improvement in residential care facilities (such as a focus on sustainable culture change).
